# Triplex real-time PCR–an improved method to detect a wide spectrum of mitochondrial DNA deletions in single cells

**DOI:** 10.1038/srep09906

**Published:** 2015-05-19

**Authors:** Karolina A. Rygiel, John P. Grady, Robert W. Taylor, Helen A. L. Tuppen, Doug M. Turnbull

**Affiliations:** 1 Wellcome Trust Centre for Mitochondrial Research and Newcastle University Centre for Ageing and Vitality, Institute of Neuroscience, Newcastle University, Newcastle upon Tyne, NE2 4HH, UK

## Abstract

Mitochondrial DNA (mtDNA) mutations are commonly found in the skeletal muscle of patients with mitochondrial disease, inflammatory myopathies and sarcopenia. The majority of these mutations are mtDNA deletions, which accumulate to high levels in individual muscle fibres causing a respiratory defect. Most mtDNA deletions are major arc deletions with breakpoints located between the origin of light strand (O_L_) and heavy strand (O_H_) replication within the major arc. However, under certain disease conditions, rarer, minor arc deletions are detected. Currently, there are few techniques which would allow the detection and quantification of both types of mtDNA deletions in single muscle fibres. We have designed a novel triplex real-time PCR assay which simultaneously amplifies the *MT-ND4* gene in the major arc, the *MT-ND1* gene in the minor arc, and the non-coding D-Loop region. We demonstrate that this assay is a highly sensitive and reliable tool for the detection and quantification of a broad range of major and minor arc mtDNA deletions with the potential to investigate the molecular pathogenesis in both research and diagnostic settings.

Mitochondria contain their own genome–a maternally-inherited 16.6 kb circular double-stranded DNA molecule (mtDNA) encoding 13 proteins, 22 mt-tRNAs and two mt-rRNAs. Each mitochondrion harbours multiple copies of mtDNA and each cell contains multiple mitochondria. Point mutations and large-scale rearrangements (deletions or duplications) of the mitochondrial genome are associated with a wide-range of human clinical presentations^1^. In the majority of cases the mtDNA mutations are heteroplasmic (a mixture of mutated and wild type mtDNA) and the percentage of mutated to total mtDNA copies is referred to as mutation load. Each tissue exhibits a different mutational threshold which, when reached, leads to respiratory deficiency which is often demonstrated by the histochemical defect of cytochrome *c* oxidase (COX).

MtDNA deletions are frequently reported in disease and ageing^2^,^3^,^4^,^5^. Around 20% of patients suffering from mitochondrial disease have a single large-scale mtDNA deletion^4^. Large-scale mtDNA deletions are also the major cause of respiratory deficiency in a number of ageing post-mitotic tissues including brain and muscle^6^,^7^. The majority of mtDNA deletions reported to date occur within the major arc of the mitochondrial genome^8^. However, a growing number of studies, mostly originating from ageing research, have identified unusual deletions spanning beyond the origin of light strand replication (O_L_) into the minor arc^9^,^10^,^11^. One explanation for why the major arc deletions occur with a much higher frequency may be the mechanism of mtDNA replication^12^. According to the canonical strand-displacement model , both origins of replication (O_H_ and O_L_) are essential for mtDNA duplication^13^. Alternative replication models, both strand-coupled synthesis and a model involving a major D-loop replication origin, postulate that O_L_ is dispensable^14^,^15^.

The gold standard technique for the detection and quantitative assessment of mtDNA deletions is Southern blotting; however, this requires large quantities of DNA and is therefore not suitable for small samples such as single cell preparations^16^. In both ageing and a variety of disease pathologies, mtDNA deletions accumulate to high levels in individual cells, but affect only a minor proportion of mtDNA molecules in tissue homogenate. Therefore the only reliable quantification methods are PCR-based and performed on DNA extracted from single cells^16^. A three primer real-time PCR assay has been historically used to quantify the level of a specific mtDNA deletion in *trans*mitochondrial cybrid cells^17^. This method relies on a pair of probes positioned between a forward and one of two reverse primers. As one of the probes is located within the deleted region and the other within the preserved region of the mtDNA, the ratio of mutated to wild-type mtDNA copies (deletion load) can be determined. This assay quantifies a specific mtDNA deletion of known breakpoints but cannot be used for identification of novel mtDNA deletions. An alternative method, developed in our laboratory, uses TaqMan real-time PCR chemistry to perform a simultaneous analysis of two mitochondrial genes; *MT-ND1* that is rarely deleted and *MT-ND4* which is often deleted as the majority of deletions appear within the major arc^18^,^19^,^20^. The detected level of *MT-ND1* indicates the total amount of mtDNA present in the sample, whilst the relative level of *MT-ND4* allows quantification of the proportion of the deleted species. Finally, an additional TaqMan real-time PCR multiplex assay, which allows quantification of deletions as well as mtDNA copy number by measuring two mitochondrial targets (*MT-ND4* and D-Loop region) and a nuclear gene (*β2M*) has recently been described^21^. Similar to the previous technique however, this approach is limited to the detection of major arc deletions.

Although our duplex real-time PCR assay is a useful tool for quantifying major arc deletions of the mitochondrial genome, it fails to detect and quantify rarer mtDNA deletions which extend into the minor arc deleting or partially deleting both *MT-ND1* and *MT-ND4*. In order to facilitate analysis of a much broader spectrum of mtDNA deletions we have developed a new assay which simultaneously amplifies part of the D-Loop, *MT-ND1* and *MT-ND4*. The D-Loop target is positioned within the non-coding region (NCR) of mtDNA, which has been shown to be conserved in 98.8% of mtDNA molecules reported to harbour a mtDNA deletion^2^. Here we show that this triplex real-time PCR assay can accurately quantify mtDNA deletion load in single cells from both ageing and disease tissues.

## Materials and Methods

### Samples

DNA samples from a variety of different tissues were used in this study including whole blood from healthy, young individuals (n = 3), muscle homogenate from a young control (n = 1) and single large-scale mtDNA deletion patients (SD; n = 2), and single muscle fibres from sporadic inclusion body myositis patients (sIBM) (n = 4) and SD patients (n = 2). We used SD samples as they had well characterised major arc deletions and sIBM samples as they encompass unusual mtDNA rearrangements^2^. All single muscle fibres were laser microdissected from tissue sections using the PALM system (Zeiss) and lysed in Tris/Tween/Proteinase K lysis buffer (0.5 M Tris-HCl, 0.5% Tween 20, 1% Proteinase K, pH 8.5). The majority of the dissected muscle fibres were respiratory-deficient (COX-deficient) and three respiratory-normal (COX-normal) cells from a heathy control were included as a positive control.

Ethical approval was granted from Newcastle and North Tyneside LREC. The experiments were carried out in accordance with the approved guidelines. An informed consent was obtained from all subjects taking part in this study.

### Plasmid p7D1

**Figure 1:**
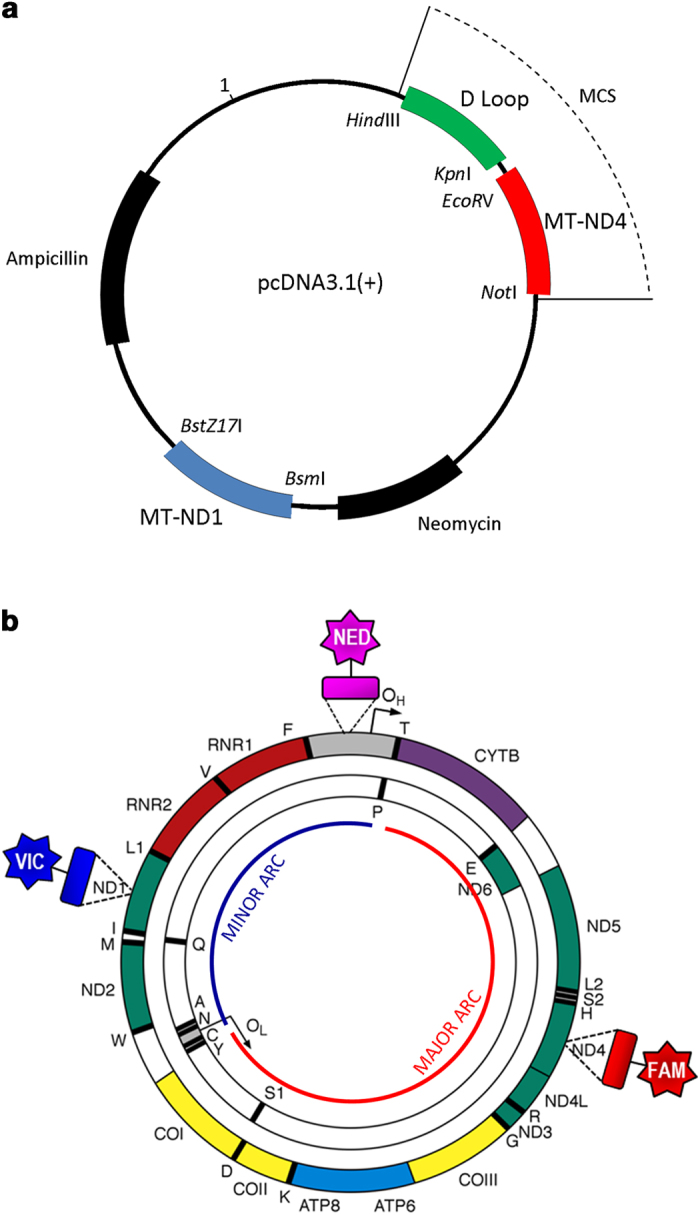
A circular map of the p7D1 plasmid vector. Restriction sites used for cloning in the three targets: *MT-ND1*, *MT-ND4* and D-Loop, are depicted in italics. MCS: multiple cloning site (**a**). A circular map of the human mitochondrial genome. The locations of TaqMan probes are depicted: VIC (recognising *MT-ND1*), FAM (recognising *MT-ND4*) and NED (recognising D-Loop region) (**b**).

In order to obtain a template for standard curve generation a plasmid construct was engineered as follows. A portion of the *MT-ND4* gene (605 bp) was amplified with the forward primer 5′-TGTAAAACTGCGGCCGCTCTCCCTCTACATATTTACCAC-3′ and the reverse primer 5′-CATGCGGCCGCTATGACCGTGGCTCAGTGTCAGTTCG-3′. The amplicon was sequentially digested with *BsrB*I and *Not*I and the resulting 580 bp fragment was inserted into the *EcoR*V and *Not*I restriction sites of the pcDNA3.1( + ) vector (Life Technologies) multiple cloning site. The forward and reverse primers 5′-GCAATGGCATTCCTAATGCTT-3′ and 5′-CAGGAATGCATCATGACCAGAGTGCGTCATATGTTGTTC-3′ were used to amplify a region of the *MT-ND1* gene (724 bp). This PCR product was cut with *Bsm*I and *Psi*I (642 bp) and inserted into the *MT-ND4* pcDNA3.1( + ) vector, digested with *Bsm*I and *BstZ17*I. Finally, a 634 bp sequence of the mitochondrial D-Loop was amplified with the forward primer 5′-TATCGAAGCTTCAACCCTCAACTATCACA-3′ and reverse primer 5′GTATCGGGTACCTGTGCAGACATTCAATTGTTA-3′. After digestion with *Kpn*I and *Hind*III, the 621 bp product was inserted into the *Kpn*I and *Hind*III restriction sites of the *MT-ND4*/*MT-ND1* pcDNA3.1( + ) vector multiple cloning site (Fig. 1a). The resultant p7D1 plasmid (7,194 bp) was verified as having only a single copy of each insert by Sanger sequencing.

### Triplex real-time PCR assay design

The real-time PCR assay was performed using TaqMan chemistry. The primers to detect *MT-ND1* and *MT-ND4* have been previously reported^19^. The primers/probe to detect the D-Loop region were designed using Primer3 software (Whitehead Institute for Biotechnology Information, Bethesda, USA). Primers amplifying a fragment of D-Loop were: Forward 5′-CCCACACGTTCCCCTTAAATAA-3′ (np 16536-16557) and Reverse 5′-CGTGAGTGGTTAATAGGGTGATAGAC-3′ (np 34-9). These primers were screened for common population polymorphisms using the “mtDNA Population Database”^22^. Probes were coupled with a non-fluorescent quencher (TaqMan MGB probes) and their sequences were: *MT-ND1* VIC-5′-CCATCACCCTCTACATCACCGCCC-3′-MGB (np 3506-3529), *MT-ND4* FAM-5′-CCGACATCATTACCGGGTTTTCCTCTTG-3′-MGB (np 12111-12138), D-Loop NED-5′-ACATCACGATGGATCAC-3′-MGB (np 16559–6) (Fig. 1b). All primer and probe locations are indicated according to the revised Cambridge Reference Sequence (rCRS); GenBank accession number NC_012920.1. PCR amplification was carried out in 25 μl reactions and at least 3 replicates were used for each sample. Each plate contained a range of serial dilutions of either a control DNA template or plasmid p7D1 for standard curve generation. TaqMan Universal Master Mix was used and primers were diluted to 0.3 μM and probes to 0.1 μM final concentration. A range of experiments were carried out to test inter- and intra-plate variability. The real-time PCR amplification was performed on a StepOnePlus thermo cycler (Applied Biosystems) using the following thermal profile: 50 °C for 2 min, 95 °C for 10 min, 40 cycles of 95 °C for 15 sec, 60 °C for 1 min. The results were analysed with StepOne software v.2.0 (Life Technologies). The ratios of *MT-ND1*/D-Loop, *MT-ND4*/D-Loop and *MT-ND4/MT-ND1* were calculated using standard curves.

### Validity of the assay

In order to validate the assay, five serial dilutions of young control DNA (concentration of 62.5 ng/μl based on Nanodrop measurement) extracted from a muscle homogenate (which we refer to as “standard”) were tested both in a single real-time PCR reaction, where just one target (a region of the D-Loop, *MT-ND1* or *MT-ND4*) was amplified (singleplex), and in a reaction where the D-Loop, *MT-ND1* and *MT-ND4* were amplified simultaneously (triplex). All reactions were conducted on the same plate. The amplification curves were examined for consistency, the amplification efficiencies calculated using the curves’ slope values for each target [E = (10^(−1/slope)−1)*100] and compared between singleplex and triplex conditions. Similarly, serial dilutions of plasmid p1D7 (concentration of 50 ng/μl) were also validated on a separate plate in a triplex reaction, and amplification efficiencies were calculated in the same way.

### Repeatability and reproducibility

Serial dilutions of the standard DNA were analysed in triplicate in eight separate experiments and amplification efficiencies were calculated in order to assess reproducibility across independent plates. In addition, three normal control DNA samples were quantified in a minimum of six independent experiments and *MT-ND1*/D-Loop, *MT-ND4*/D-Loop and *MT-ND4*/*MT-ND1* ratios were compared between experiments. This was performed to ensure that neither the differences within the same sample examined multiple times, nor between the three healthy control samples, were significant. Furthermore, two DNA samples of equal concentration and known mtDNA deletion levels (control DNA: 0% mtDNA deletion and SD patient DNA: 75% mtDNA deletion located in the major arc) were mixed in different ratios (100% control DNA, 1:9, 1:4, 1:2.3, 1:1.5, 1:1, 1.5:1, 2.3:1, 4:1, 9:1, 100% deleted DNA) in sextuplicate to test precision and accuracy of the assay. Finally, a set of single cell DNA samples (n = 40) from two patients with single mtDNA deletions and four sIBM patients (n = 60) were tested for mtDNA deletion load to verify whether this assay was sensitive enough for single cell analysis. Ratios are presented as a percentage. For example, an *MT-ND4*/*MT-ND1* ratio of 90% signifies that *MT-ND4* levels are 90% of *MT-ND1* levels, in other words 10% of mtDNA molecules tested are harbouring an *MT-ND4* deletion.

### Long range PCR

DNA samples from selected single cells used in the triplex assay were amplified using a long range PCR protocol and 130 F (np 130–161) and 16382 R (np 16361–16382) oligonucleotide primers. PCR reactions were carried out using Prime Star GXL polymerase kit (Clontech) and the cycling conditions were: 35 cycles of 98 °C for 10 sec and 68 °C for 11 min. Amplified products were separated through 0.7% agarose gels and imaged using ChemiDoc (Biorad).

### Ion Torrent sequencing

We used deep sequencing to characterise mtDNA deletion breakpoints. Briefly, selected amplicons ( ≤ 9 kb), were purified using Agencourt AMPure XP reagent and assessed for concentration and quality using an Agilent 12000 DNA kit on a Bioanalyzer (Agilent 2100, Agilent Technologies). 100 ng of each DNA sample processed using the Ion Xpress Plus Fragment Library Kit (Life Technologies) according to manufacturer’s instructions. The samples were fragmented using Ion Shear Plus Enzyme, then ligated to barcoded adapters. Fragments ~330 bp in length were recovered with E-gel Size Select 2% gels (Life Technologies). The libraries were then amplified and assessed on a Bioanalyzer using the Agilent High Sensitivity DNA Kit. The libraries were pooled in equimolar ratios (final concentration 26 pM), then clonally amplified onto Ion Sphere Particles using the Ion OneTouch system and the Ion OneTouch 200 Template kit v2. Subsequently, the Ion OneTouch ES Instrument allowed enrichment of template-positive Spheres using magnetic bead technology. The enriched samples were loaded onto an Ion 316v2 Chip for deep sequencing on an Ion Torrent system. Data were analysed using the Torrent Browser (v4.0.2) and alignments to the human mtDNA reference sequence were interpreted using IGV (version 2.1; Broad Institute).

### Statistical analyses

For each ratio under study, the distribution of the ratio values for control samples was both examined visually and tested for normality using three common tests (Shapiro-Wilk, Kolmogorov-Smirnov, and Anderson-Darling)^23^. As the distribution of all the control sample ratios passed all normality tests and demonstrated no obvious deviation through visual inspection, mean and standard deviations were calculated for each ratio, which were used to determine 95% confidence intervals. SAS version 9.2 (Cary, NC) was used for all analyses.

## Results

### Absence of interactions between real-time reactions

**Figure 2:**
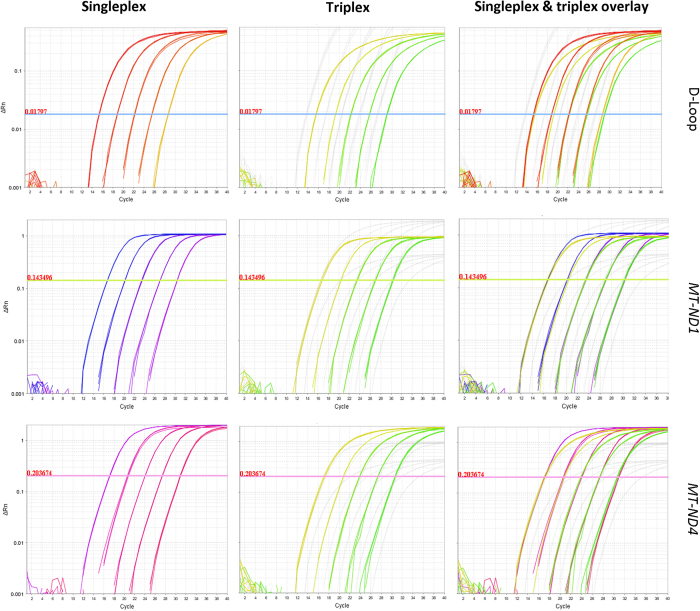
Real-Time PCR amplification plots of D-Loop using five serial dilutions of standard DNA. The assay was performed as a singleplex reaction (D-Loop or *MT-ND1* or *MT-ND4* target only) or a triplex reaction (D-Loop, *MT-ND1* and *MT-ND4*) to investigate the potential interaction between the primers. Overlapping amplification curves in the singleplex and triplex reactions did not identify any inhibitory effect.

**Figure 3:**
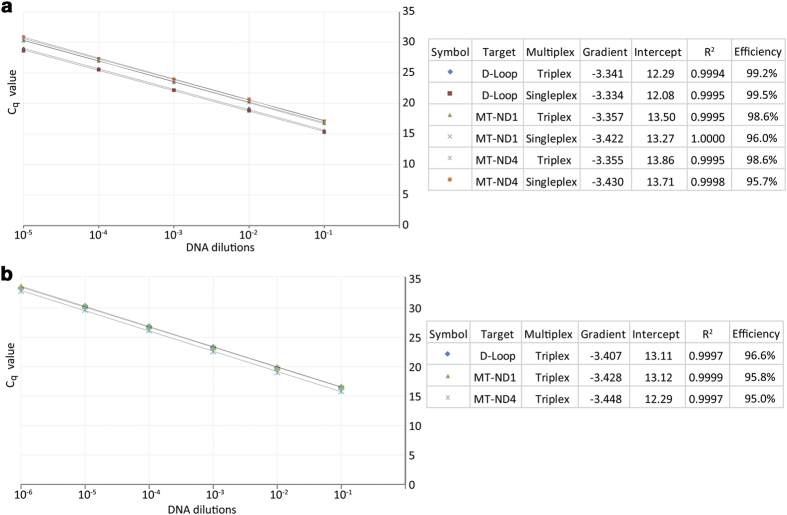
Efficiencies for standard DNA standard curves analysed as singleplex and triplex reactions were almost 100% and were very similar to each other (**a**). Standard curves obtained with plasmid p7D1 and analysed in a triplex reaction had similar efficiencies (around 95%) (**c**). Cycle quantification (C_q_) values were plotted on the y axis and serial ten-fold dilutions of the DNA template (10^−1^, 10^−2^, …) on the x axis (**a**,**b**). Typical C_q_ values for single cell preparations fell between 26 and 33. Outlying samples with higher C_q_ values were not used for the downstream analyses.

We conducted singleplex and triplex real-time reactions with all three targets, *MT-ND1*, *MT-ND4* and D-Loop, using serially-diluted standard DNA. The amplification curves for each mtDNA target from the singleplex and triplex reactions overlapped (Fig. 2), confirming that the simultaneous amplification of all three targets does not limit the amplification of any individual target. The reaction efficiencies calculated using the standard curves were close to 100% for all three targets and there was no difference between singleplex and multiplex reactions (Fig. 3a). We performed the same experiment using the plasmid p7D1 DNA, which contains sequences of all three targets at 1:1:1 ratio. The efficiencies of the real-time reactions were again close to 100% and all targets amplified equally well (Fig. 3b), confirming that none of the primer pairs used limited amplification of the other targets.

### Repeatability and reproducibility of the triplex assay

**Figure 4:**
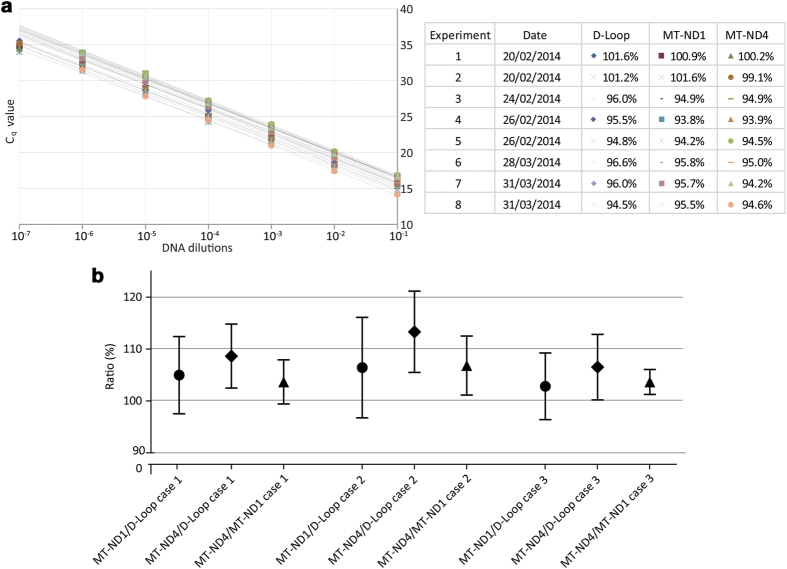
Standard curves obtained using standard DNA showed strong consistency in eight separate experiments. Real-Time PCR efficiencies varied between 100 and 94% and all three targets (*MT-ND1*, *MT-ND4* and D-Loop) amplified with almost identical efficiency within each experiment (**a**). Three healthy young control DNA samples (case 1–3) were tested in six or nine independent reactions. Standard DNA was used for standard curve generation. Means of *MT-ND1*/D-Loop, *MT-ND4*/D-Loop and *MT-ND4*/*MT-ND1* ratios were just above 100% (102.8–113.3%) and the standard deviation (sd) not greater than 10%. Samples used were diluted to concentrations similar to that of single cells (C_q_ between 27 and 29) (**b**).

**Figure 5:**
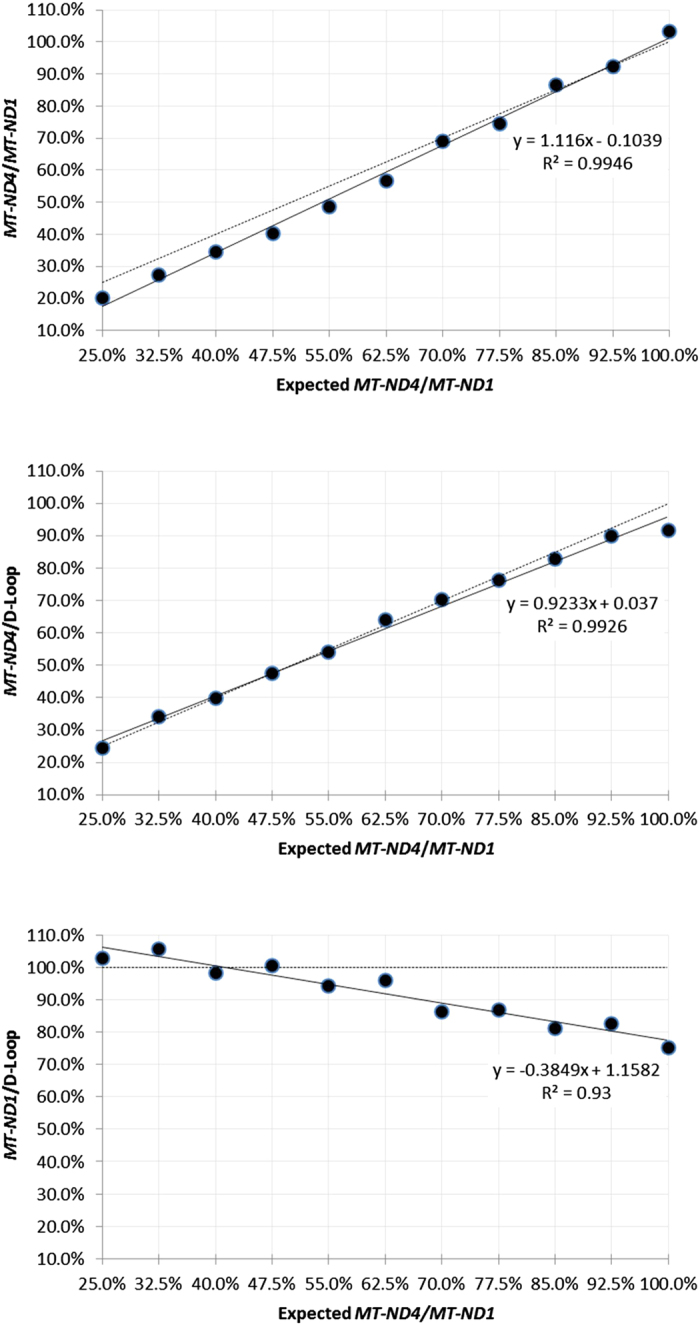
DNA from a healthy control (*MT-ND4*/*MT-ND1* = 100%) and a single deletion patient (*MT-ND4*/*MT-ND1* = 25%) were mixed in different proportions (the dilution factor was constant). Eleven samples ranging from 25% to 100% of *MT-ND4* levels were analysed in a triplex assay. The observed heteroplasmy levels were plotted on the graphs. The dotted line on each graph indicates the predicted outcome of the experiment if the D-loop was double-stranded in both the deleted and wild-type DNA samples. The mixed samples amplified at C_q_ of approximately 25.

Eight independent experiments were performed using at least six 10-fold dilutions of the standard DNA. The reaction efficiencies were very similar for all three targets within each triplex assay and varied by a maximum of 8% between different experiments (Fig. 4a). In order to investigate whether the assay generated reliable and reproducible results, we tested three DNA samples extracted from whole blood of healthy young subjects in a minimum of six independent experiments. Analysis of the results was carried out using a standard curve of the standard DNA. The respective means ± standard deviations of *MT-ND1*/D-Loop, *MT-ND4*/D-Loop and *MT-ND4*/*MT-ND1* ratios were: 105.0 ± 7.4, 108.6 ± 6.2, 103.7 ± 4.3 (case 1); 106.4 ± 9.7, 113.3 ± 7.8, 106.8 ± 5.7 (case 2); 102.8 ± 6.4, 106.5 ± 6.3, 103.7 ± 2.4 (case 3) (Fig. 4b). The ratios signify percentage level of a target relative to either D-Loop or *MT-ND1*. We then quantified the precision of the assay. Two DNA samples from whole blood and muscle homogenate with different mtDNA deletion levels, but of the same mtDNA concentration, were mixed in several set proportions. As the mtDNA concentrations were equal, the *MT-ND4*/*MT-ND1* and *MT-ND4*/D-Loop ratios were expected to increase linearly from 25% to 100% across the sample series, whilst *MT-ND1*/D-Loop ratios were expected to remain constant at 100%. The *MT-ND4*/*MT-ND1* and *MT-ND4*/D-Loop ratios did perform as predicted, exhibiting a direct linear relationship across the sample range (Fig. 5). However, whilst the *MT-ND4*/*MT-ND1* ratio peaked at 103.2%, the *MT-ND4*/D-Loop ratio only reached a maximum of 77.6%. Furthermore, the *MT-ND1*/D-Loop ratios progressively decreased from 102.8% in sample 1 to 75.2% in sample 11. We hypothesised that these discrepancies were a consequence of the triple-stranded nature of the D-Loop region to which the D-Loop forward primer can bind. Different levels of triple-strand formation in the two DNA samples would account for the differences in *MT-ND1*/D-Loop and *MT-ND4*/D-loop ratios observed.

### Normalisation of the data using healthy controls

To correct for the extra D-Loop binding, we established the normal ranges of the *MT-ND1*/D-Loop and *MT-ND4*/D-Loop ratios in control muscle homogenate and whole blood DNA. Normalisation of the data is achieved by dividing each ratio by the average from all normal muscle homogenate and blood DNA tested, to achieve a normalised *MT-ND1*/D-Loop and *MT-ND4*/D-Loop ratio of 100%.

Results from 29 independent experiments, obtained from 4 samples from blood and muscle homogenate were analysed using the standard DNA standard curve, and 31 independent experiments from the same samples using plasmid p7D1. Both distributions were Gaussian, thus we were able to use the mean for normalisation, and the standard deviation to define a tolerance ( ± 1.96 x standard deviations).

Using the standard DNA standard curve the normalised tolerances were ± 14.3% and ± 14.9% for *MT-ND1*/D-Loop and *MT-ND4*/D-Loop ratios respectively; for the plasmid p7D1 standard curve the normalised tolerances were ± 14.5% and ± 10.6% respectively (Table 1).

Using plasmid p7D1 standard curve, normalisation is achieved by dividing the *MT-ND1*/D-Loop and *MT-ND4*/D-Loop ratios by 0.863 and 0.847 respectively (mean ratios obtained from healthy control samples, Table 1). Similarly the normalisation factors using the standard DNA standard curve are 1.054 and 1.087 respectively (Table 1). *MT-ND4*/*MT-ND1* does not require normalisation as this is not affected by the triple stranded D-Loop.

### The assay is sensitive in detecting mtDNA deletions in individual cells

DNA extracted from muscle homogenate and DNA from single, laser-microdissected muscle fibres were obtained from two SD cases (SD1 and SD2). Both patients had previously characterised major arc deletions: SD1 harbouring a 8704 bp mtDNA deletion (breakpoints: np 7175 and np 15879) and SD2 a 4113 bp deletion (breakpoints: np 11262 and np 15375). *MT-ND1*/*MT-ND4*/D-Loop ratios were calculated using standard DNA standard curve and normalised. The triplex assay demonstrated *MT-ND4* deletion in homogenate DNA from both cases (65% and 30% of *MT-ND4* levels preserved in SD1 and SD2 respectively), whereas the *MT-ND1* site was entirely preserved (around 100% *MT-ND1*/D-Loop ratio) (Table 2). Over 40 single COX-deficient muscle fibres were tested and all of them demonstrated a clonally-expanded *MT-ND4* deletion (Table 2 contains representative results from six cells per case). The heteroplasmy levels differed between individual cells and between the two cases. Single cells from SD1 exhibited *MT-ND4* levels of between 10% and 18% compared to D-Loop and *MT-ND1*; cells from SD2 varied between 1% and 5%. None of the cells harboured an *MT-ND1* deletion.

### sIBM muscle exhibits a spectrum of mtDNA deletions

We then investigated mtDNA deletions in single respiratory-deficient (COX-deficient) cells and respiratory-normal (COX + ve) cells (serving as endogenous control) from four sIBM cases. The data were analysed in 60 cells using standard DNA and/or plasmid p7D1 standard curves and normalised. Representative results are shown in Table 3. Some respiratory-deficient fibres did not have any detectable levels of deletion of either *MT-ND1* or *MT-ND4*, mimicking the results of respiratory-normal counterparts. A range of mtDNA deletions were detected in other COX-deficient single fibres from sIBM patients. The majority of cells had mtDNA deletions encompassing *MT-ND4* only, but we also identified cells with reduced levels of either *MT-ND1* or both *MT-ND1* and *MT-ND4* (Table 3). These unusual deletions were found in all four sIBM patients with varying frequencies. Importantly, for those samples analysed using both standard curves (standard DNA and plasmid DNA) the two approaches yielded consistent results.

### Long range PCR and breakpoint sequencing analyses validates the triplex assay

**Figure 6:**
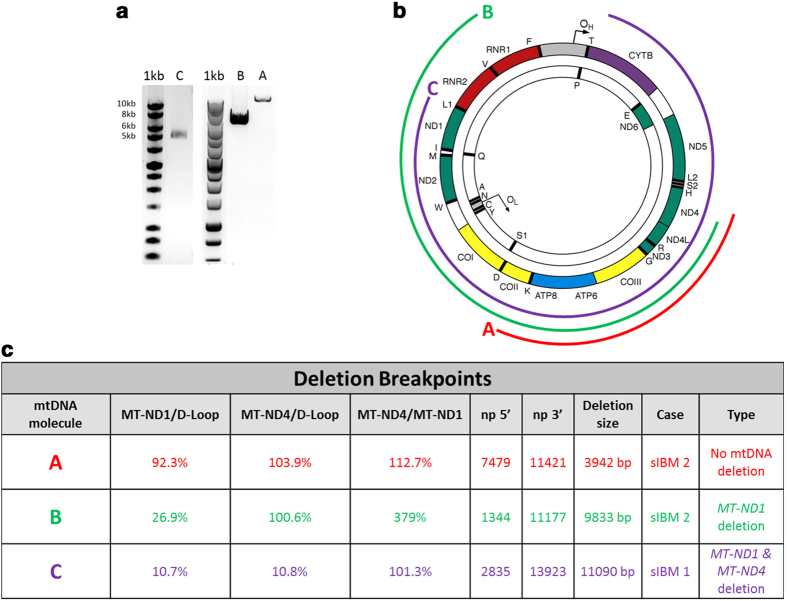
Validation of the triplex assay by long range PCR and deep sequencing of selected single cell DNA samples. Long range PCR amplimers obtained from three cells (**a**–**c**) from two sIBM patients (sIBM 1 and sIBM 2) separated through a 0.7% agarose gel. Sizes are indicated by 1 kb DNA ladder on the left (**a**). Location of identified mtDNA deletions depicted by coloured curved lines around the mitochondrial genome. The left and right ends of each line demonstrate the position of the 5′ and 3′ breakpoints respectively (**b**). A table showing the ratios of *MT-ND1*/D-Loop, *MT-ND4*/D-Loop and *MT-ND4*/*MT-ND1* together with the exact location of the mtDNA deletion breakpoints (**c**).

In order to further validate the triplex assay, we selected a representative group of single cell preparations (a cell with no detectable deletion, *MT-ND1* deletion and combined *MT-ND1* + *MT-ND4* deletion) and subjected them to long range PCR amplification. The primers were chosen to amplify almost the entire wild type mitochondrial genome (~16.5 kb). As expected, the largest PCR product was derived from a cell devoid of mtDNA deletions (cell A), a band of approximately 7 kb was amplified from a cell with *MT-ND1* (cell B) deletion and the smallest (~5 kb) deletion was detected in a cell harbouring *MT-ND1* + *MT-ND4* deletion (cell C) (Fig. 6a). Deep sequencing of the PCR products revealed results fully consistent with the triplex PCR data obtained for cells B and C; 9.8 kb and 11 kb mtDNA deletions were detected in these cells respectively (Fig. 6c). A short deletion of 3.9 kb, encompassing neither *MT-ND1* nor *MT-ND4*, was detected in cell A. It was missed by the triplex assay analysis as its 3′ breakpoint was located upstream of the *MT-ND4* primers binding sites (Fig. 6b, c).

## Discussion

The purpose of this study was to design an assay allowing detection and quantification of the great majority of mtDNA deletions in single cells. The *MT-ND1*/*MT-ND4* real-time PCR duplex assay developed by our group^18^,^19^ is not suitable for the detection of rarer mtDNA deletions, involving regions outside the major arc. As a growing number of studies examining different diseases are reporting large-scale deletions removing O_L_ and extending far into the minor arc, it is very important to develop a tool to easily discern and quantify these deletions in cells and tissues. An in-depth review of mtDNA rearrangements by Damas *et al.* demonstrates that the most conserved region of mtDNA (preserved in 98.8% of mtDNA molecules in which deletions have been reported to date) is the non-coding control region, containing the D-loop^2^. Therefore we decided to design a triplex TaqMan assay which targets the D-Loop along with the other two mtDNA targets, *MT-N*D1 and *MT-ND4*. The D-Loop region has been previously used as a target in a multiplex assay in conjunction with *MT-ND4* allowing detection of major arc deletions only^21^.

One of the challenges of developing a multiplex real-time PCR assay is avoiding non-specific interactions between primers amplifying different DNA targets. Therefore it is essential to directly compare amplification efficiencies of each target tested both individually and in the multiplex reaction. There was no inhibitory effect of any of the three targets (*MT-ND1*, *MT-ND4* and D-Loop) as they all amplified with the same efficiency in singleplex and triplex reactions.

The D-Loop target used in this assay binds to the non-coding region (NCR) of the mitochondrial genome. The NCR spans approximately 1.1 kb between nucleotide positions 16024 and 576^24^. A large part of the NCR constitutes a triple-stranded fragment^25^, which starts around O_H_ and terminates just after termination-associated sequence (TAS)^26^. This extra strand, called 7S DNA, is approximately 650 nucleotides in length and forms a stable D-Loop structure^27^. The number of mtDNA molecules containing 7S DNA can vary over time and, more importantly, different cell types have different proportions of 7S DNA-containing molecules (10% of molecules in cultured human cells and 90% of *Xenopus* oocytes)^27^. The more metabolically active the cells are, the more 7S DNA their mitochondrial genomes will possess^26^.

Primers targeting the triple-stranded region will thus bind to both the H-strand and 7S DNA if it is present, which will perturb the ratio of the D-Loop binding compared to other mtDNA targets. To avoid this, we attempted to design primers binding to the region between tRNA^Phe^ and LSP–the fragment of NCR not associated with RNA, RNA/DNA hybrid or 7S DNA. However, it was not feasible to find an optimal primer pair in this short sequence of approximately 130 nucleotides.

Nevertheless, we showed that by quantifying the variability in the D-Loop ratios from controls it was possible to normalise the data obtained using the D-Loop primers/probe. Our data demonstrate that such normalisation was sufficient to enable an accurate assessment of the deletion load in all samples. This step was not included in the previous study despite the D-Loop primers binding within the triple stranded region of the NCR^21^. The use of the plasmid p7D1 containing one copy of all three targets per molecule made our normalisation precise and the assay sensitive and quantitative. It is, however, advisable to perform a normalisation step for all patient groups tested in order to quantify the normal (not deleted) range of the *MT-ND1*/D-Loop and *MT-ND4*/D-Loop ratios, as the amount of D-Loop could potentially be influenced by disease. The latter would involve scaling the data by the mean amount of D-Loop in healthy cells (COX-normal) from several cases of the same disease.

We tested the triplex real-time assay on a range of samples (homogenate and single cell DNA) from single deletion patients and sIBM, and we obtained reliable and reproducible results. As confirmed before by sequencing of the mtDNA deletion breakpoints, both SD patients’ muscle homogenate tested in this study contained major arc deletions, which were detected with *MT-ND4*/D-loop and *MT-ND4*/*MT-ND1* ratios in SD1 (34.79% and 33.63% mtDNA deletion respectively) and SD2 (70.45% and 70.41% respectively). Single laser-microdissected COX-deficient muscle fibres did not differ by more than 0.6% between *MT-ND4*/D-loop and *MT-ND4*/*MT-ND1*. In COX-deficient cells from sIBM cases we detected some without mtDNA deletions, some with major arc deletions removing only the *MT-ND4* gene, some with minor arc deletions removing both *MT-ND1* and *MT-ND4* genes, and some with deletions removing *MT-ND1* but preserving *MT-ND4*. Long range PCR and deep sequencing of mtDNA obtained from a representative group of three single cells with isolated *MT-ND1*, combined *MT-ND1 + MT-ND4* deletion and “no deletion” confirmed the expected locations of deletion. Interestingly, the cell, in which no deletion had been detected using the triplex assay, harboured a short mtDNA deletion which only encompassed a proportion of the *MT-ND4* gene. The identification of such a variety of mtDNA deletions in sIBM is in agreement with previously published reports from inflammatory myopathies^11^ as well as currently unpublished data from our laboratory. These data also provided evidence that a marked proportion of mtDNA deletions occurring in disease muscle samples would not be detected using the standard duplex real-time PCR assay. There are still small deletions, or deletions of the minor arc, whose 5′ breakpoint is situated within *MT-ND2* gene which will be missed using our assay. This highlights the importance of developing new multiplex PCR-based techniques to permit the detection of other, currently undetectable, mtDNA deletions in single cells. In summary, we have shown that the addition of a D-Loop target to the established *MT-ND4*/*MT-ND1* assay allows quantification of minor arc mtDNA deletions, providing the opportunity for more comprehensive studies into the role of mtDNA deletions in disease. Unlike the previously published real-time PCR assay incorporating the D-Loop target^21^ our method corrects for the amount of triple-stranded NCR improving reliability and quantitation. We believe that this novel triplex real-time PCR assay will prove to be a vital tool in both research and diagnostic settings for identifying and quantifying the majority of mtDNA deletions occurring in disease and ageing.

## Author Contributions

K.A.R. carried out the experimental work and wrote the majority of the manuscript with contributions from J.P.G. and H.A.L.T. J.P.G. performed the statistical analysis. H.A.L.T. constructed the p7D1 plasmid and advised on many aspects of the methodology. J.P.G., H.A.L.T., D.M.T. and R.W.T. read and reviewed the manuscript.

## Additional Information

**How to cite this article**: Rygiel, K. A. *et al*. Triplex Real-Time PCR - an improved method to detect a wide spectrum of mitochondrial DNA deletions in single cells. *Sci. Rep.* **5**, 9906; doi: 10.1038/srep09906 (2015).

## Figures and Tables

**Table 1:** Normalisation of the triplex assay results using healthy full blood and muscle homogenate DNA samples.

**Source**	**Ratio**	**Mean**	**SD**	**N**	**Unnormalised Tolerance 1.96 x SD**	**Normalised SD**	**Normalised Tolerance**
Standard DNA	*MT-ND1*/D-loop	1.054	0.077	29	0.151	0.073	0.143
Standard DNA	*MT-ND4*/D-loop	1.087	0.083	29	0.163	0.076	0.149
Standard DNA	*MT-ND4*/*MT-ND1*	1.032	0.061	29	0.120	0.059	0.116
Plasmid p7D1	*MT-ND1*/D-loop	0.836	0.062	31	0.122	0.074	0.145
Plasmid p7D1	*MT-ND4*/D-loop	0.847	0.046	31	0.090	0.054	0.106
Plasmid p7D1	*MT-ND4*/*MT-ND1*	1.011	0.039	31	0.076	0.039	0.076

**Table 2:** Muscle homogenate and single cell DNA from two single mtDNA deletion patients (SD1 and SD2) with a deletion in the major arc were tested using the triplex assay.

**Case**	**Sample**	***MT-ND1*****/D-loop**	***MT-ND4*****/D-loop**	***MT-ND4*****/*****MT-ND1***
SD1	Homogenate	101.4%	65.2%	66.4%
SD1	Single Cell	106.3%	17.8%	17.2%
SD1	Single Cell	103.7%	9.8%	9.7%
SD1	Single Cell	104.8%	14.0%	13.8%
SD1	Single Cell	97.5%	11.0%	11.6%
SD1	Single Cell	102.3%	13.9%	14.0%
SD1	Single Cell	105.7%	11.4%	11.1%
SD2	Homogenate	103.0%	29.6%	29.6%
SD2	Single Cell	102.2%	5.1%	5.1%
SD2	Single Cell	102.5%	2.4%	2.4%
SD2	Single Cell	101.5%	3.2%	3.3%
SD2	Single Cell	100.5%	1.5%	1.6%
SD2	Single Cell	103.9%	2.2%	2.2%
SD2	Single Cell	106.3%	4.9%	4.7%

Standard DNA was used for standard curve generation. All single fibres were respiratory-deficient (COX-deficient). The ratios of *MT-ND1*/D-Loop, *MT-ND4*/D-Loop and *MT-ND4*/*MT-ND1* are depicted in the Table.

**Table 3:** Single respiratory-deficient muscle fibres from four sporadic inclusion body myositis (sIBM) patients were tested using the triplex assay with either standard DNA or plasmid p7D1 or both.
